# CD107a^+^ (LAMP-1) Cytotoxic CD8^+^ T-Cells in Lupus Nephritis Patients

**DOI:** 10.3389/fmed.2021.556776

**Published:** 2021-03-23

**Authors:** Anika Wiechmann, Benjamin Wilde, Bartosz Tyczynski, Kerstin Amann, Wayel H. Abdulahad, Andreas Kribben, Karl Sebastian Lang, Oliver Witzke, Sebastian Dolff

**Affiliations:** ^1^Department of Nephrology, University Duisburg-Essen, Essen, Germany; ^2^Department of Medical Intensive Care I, University Hospital Essen, University Duisburg-Essen, Essen, Germany; ^3^Department of Nephropathology, University Hospital Erlangen, Friedrich-Alexander-University Erlangen-Nürnberg, Erlangen, Germany; ^4^Department of Rheumatology and Clinical Immunology, University Medical Center Groningen, University of Groningen, Groningen, Netherlands; ^5^Department of Pathology and Medical Biology, University Medical Center Groningen, University of Groningen, Groningen, Netherlands; ^6^Institute of Immunology, Medical Faculty, University of Duisburg-Essen, Essen, Germany; ^7^Department of Infectious Diseases, West German Centre of Infectious Diseases, University Duisburg-Essen, Essen, Germany

**Keywords:** cytotoxic T-cells, CD107a, LAMP-1, lupus nephritis, SLE

## Abstract

Cytotoxic CD8^+^ T-cells play a pivotal role in the pathogenesis of systemic lupus erythematosus (SLE). The aim of this study was to investigate the role of CD107a (LAMP-1) on cytotoxic CD8^+^ T-cells in SLE-patients in particular with lupus nephritis. Peripheral blood of SLE-patients (*n* = 31) and healthy controls (*n* = 21) was analyzed for the expression of CD314 and CD107a by flow cytometry. Kidney biopsies of lupus nephritis patients were investigated for the presence of CD8^+^ and C107a^+^ cells by immunohistochemistry and immunofluorescence staining. The percentages of CD107a^+^ on CD8^+^ T-cells were significantly decreased in SLE-patients as compared to healthy controls (40.2 ± 18.5% vs. 47.9 ± 15.0%, *p* = 0.02). This was even more significant in SLE-patients with inactive disease. There was a significant correlation between the percentages of CD107a^+^CD8^+^ T-cells and SLEDAI. The evaluation of lupus nephritis biopsies showed a significant number of CD107a^+^CD8^+^ T-cells mainly located in the peritubular infiltrates. The intrarenal expression of CD107a^+^ was significantly correlated with proteinuria. These results demonstrate that CD8^+^ T-cells of patients with systemic lupus erythematosus have an altered expression of CD107a which seems to be associated with disease activity. The proof of intrarenal CD107a^+^CD8^+^ suggests a role in the pathogenesis of lupus nephritis.

## Introduction

Systemic lupus erythematosus (SLE) is an autoimmune disease characterized by various organ manifestations. Inflammation of the kidney, in particular, is associated with an unfavorable prognosis (1). Although the precise pathogenesis of lupus nephritis (LN) has not been elucidated, disturbances in regulatory and effector T-cell balance seem to contribute to the development of LN (2). Despite an increasing body of evidence reporting CD4^+^ T-cell abnormalities, the role of cytotoxic CD8^+^ T-cells is less well-understood.

CD8^+^ T-cells can contribute to autoimmunity by chemokine secretion which is capable to attract other immune cells, recruitment of autoreactive CD8^+^ T-cells and killing of target cells. In SLE an increase of activated CD8^+^ T-cells expressing perforin and granzyme B has been reported (3). Interestingly, the authors found intrarenal CD8^+^ T-cells in lupus nephritis biopsies. The amount of periglomerular CD8^+^ T-cells was associated with a poor prognosis (4). This finding confirmed previous histopathological study by D'Agati et al. who reported a predominant CD8^+^ T-cell infiltrate in human lupus nephritis biopsies (5). In previous studies we proofed the hypothesis that effector T-cells migrate from peripheral blood into the kidney during active lupus nephritis and can be detected in the urine (6). The predominantly detected T-cells were CD8^+^ T-cells. The absolute cell count of urinary CD8^+^ T-cells was an excellent parameter to discriminate active from inactive lupus nephritis (7). This finding has been consistently found by Klocke et al. (8).

Despite the increasing body of evidence demonstrating the presence of kidney infiltrating cytotoxic CD8^+^ T-cells suggesting a crucial role for the renal inflammation the precise mechanism of action remain to be elucidated. CD314 (NKG2D) and CD107a (LAMP-1) are molecules expressed on activated natural killer (NK)-cells as well as on CD8^+^ T-cells. CD107a (LAMP-1) belongs to a family of highly glycosylated transmembrane proteins on human peripheral blood mononuclear cells which mediate cell adhesion to vascular endothelium which potentially enables T-cell migration into kidney (9). LAMPs may be shuttled and expressed at the cell surface after cell activation (10). Functional CD107a is required for efficient perforin delivery to lytic granules and NK-cell cytotoxicity (11).

In the present study we hypothesized that peripheral circulating cytotoxic CD8^+^ T-cells in patients with SLE have an altered CD107a expression pattern. Moreover, we aimed to analyze the cytotoxic activity of renal infiltrating T-cells reflected by CD107a expression.

## Patients and Methods

In this study 31 patients with systemic lupus erythematosus fulfilling at least 4 ACR criteria and 21 healthy controls were enrolled (12). The mean age of SLE patients was 42.5 ± 13.7 years. The mean age of healthy controls was 38.2 ± 14.4 years. Disease activity was assessed by systemic lupus erythematosus disease activity index (SLEDAI). Active disease activity was defined as SLEDAI >4, inactive disease activity was defined as SLEDAI ≤4. According to this definition 11 active and 20 inactive patients were included.

Renal involvement was defined as biopsy proven lupus nephritis. Twenty-one patients had a biopsy proven lupus nephritis which were classified according to the ISN/RPS classification from 2003. The lupus nephritis classes were class II (*n* = 3), class III (*n* = 1), class IV (*n* = 14) and class V (*n* = 3) (Table 1). All biopsies were reviewed and classified by an experienced nephropathologist (K.A.) according to the revised criteria for LN. The activity index (AI) and chronicity index (CI) were calculated for each specimen with maximum scores of 24 for the AI and 12 for the CI (13). The assessment was completed by determining the ISN/RPS 2003 classification and activity and chronicity indices for LN. For these aspects of the assessment, the definitions of the classification systems and the activity and chronicity indices were used (14).

**Table 1:** Laboratory and histological data of 10 SLE-patients with active renal disease are given.

**#**	**ISN/RPS-class^¶^**	**AI^1^**	**CI^2^**	**Haematuria**	**Proteinuria (g/24 h)**	**S-crea (mg/dl)**	**Anti-DNA-Ab (IU/ml)**
1	IV-G	10	3	+	4.2	1.07	>200
2	IV-G	7	5	++	0.6	2.31	135
3	II	1	2	+	0.6	1.30	189
4	IV-G	20	2	+++	7.0	1.35	>200
5	IV-G	8	2	-	3.2	1.67	>200
6	V	4	3	-	11.0	1.79	3.7
7	IV-G	11	1	+++	2.2	1.41	132.6
8	II	3	2	++	2.7	0.45	15
9	IV/V	16	3	+++	12.0	0.95	>200
10	n.c.	-	-	++	1.5	3.15	34

*Laboratory and histological data of 10 patients with active renal disease are shown*.

¶ *Histological ISN/RPS classification, nc, not classified*.

1 activity index (AI),

2 *chronicity index (CI)*.

Twenty-nine patients received immunosuppressive treatment. Twenty-five patients were treated with prednisone [median (range), 5 mg/d, (1–60 mg/d)]. Twenty patients received a combination of prednisone and hydroxychloroquinsulfate (*n* = 14), mycophenolate mofetil (*n* = 13), azathioprine (*n* = 4) or cyclosporine (*n* = 1). A minority was treated solely with prednisone (*n* =5) or hydroxychloroquinsulfate (*n* = 1). The study protocol was approved by the institutional review board (15-6323-BO). All patients gave informed consent for participation in this study.

### Flow Cytometry

Immunophenotyping was performed as described before (15). Briefly, 100 μl heparinized blood were mixed with antibodies: Krome Orange-conjugated anti-CD3 (clone UCHT, Beckman Coulter, Brea, USA), Pacific Blue-conjugated anti-CD8 (B9.11, Beckman Coulter, Brea, USA), Allophycocyanin (APC)-conjugated anti-CD107a (clone H4A3, Beckman Coulter, Brea, USA) and Allophycocyanin (APC)-conjugated anti-CD314 (ON72, Beckman Coulter, Brea, USA). Appropriate isotype controls were used. After vortex, all tubes were incubated for 20 min in the dark at room temperature. Next 3 ml of VersaLyse™ were added in each tube and the suspension was mixed gently with vortex. Then the tubes were incubated for 12 more minutes in the dark. Thereafter the tubes were centrifugated and the supernatant was aspirated. The cell pellet was washed with 3 ml of phosphate buffered saline (PBS). This washing step was repeated and finally 300 μl PBS were added before cells were immediately analyzed with a fluorescence activated cell sorter (FACS) NAVIOS^TM^ from Beckman Coulter. Kaluza Analysis Software (Version 1.5, Beckman Coulter) was used for analysis of flow cytometric data.

### Analysis and Scoring of Renal Biopsies

#### Immunohistochemistry

All specimens were fixed in 10% neutral buffered formalin and paraffin embedded. Five-micrometer-thick sections were deparaffinized in xylene and rehydrated in a series of different concentrations of ethanol (100, 95, 70, and 50%) (16). Tris-HCL buffer, pH 9.0, for heat-induced epitope retrieval was applied for 1 h, followed by neutralization of endogenous peroxidase with 0.3% H_2_O_2_. For CD107a staining epitope retrieval was performed with citrate buffer pH 6.0 applied for 40 min at 90°C. Protein block with 5% rabbit or goat serum in PBS for 30 min was performed. Incubation with a monoclonal mouse anti-human CD8 (clone C8/144, DAKO, Carpinteria, USA) or polyclonal rabbit anti-human CD107a (polyclonal, Bio-Rad, Munich, Germany) was performed for 60 min at room temperature. Next, sections were washed and incubated with a HRP-conjugated secondary antibody (Envison^TM^, DAKO, Carpinteria, USA) for 30 min. at room temperature. A DAB substrate (Envison^TM^, DAKO, Carpinteria, USA) was used for visualization. Washing with PBS was performed after each incubation step. Finally, the slides were counterstained with haematoxylin and mounted with Vitro-Clud® (R. Langenbrinck, Emmendingen, Germany).

Only cells with a distinctly brown and continuously stained plasma membrane were counted. Positive cells were separately counted within the interstitium and in the glomeruli. Cells with positive staining for CD8 were counted per high powerfield (40 × magnification). The average value was calculated for each biopsy.

#### Immunofluorescence Double Staining

Tissues were fixed, embedded in paraffin and sectioned as indicated above. Epitope retrieval was performed with citrate buffer pH 6.0 (Zytomed) at 90°C followed by a protein block with 5% rabbit and goat sera in PBS for 30 min at room temperature. Primary antibodies against CD107a (polyclonal rabbit, Bio-Rad) was used and incubated for 60 min followed by an incubation with a secondary antibody conjugated to FITC (Jackson Immuno). Next, primary antibody against CD8 (mouse IgG1, DAKO) was used and incubated for 60 min followed by an incubation with a secondary antibody conjugated to Cy3 (Jackson Immuno) and DAPI. Finally, the slides were mounted with ProLong® Gold antifade (Life Technologies). Tonsil sections served as positive control samples. Isotype controls for primary antibodies were used as negative controls.

#### Statistical Analysis

All values are expressed as mean ± standard deviation (SD). The significance for the differences between groups was determined by the Mann-Whitney U-test. Spearman's rank correlation was applied to detect correlations between different study parameters. Differences were considered statistically significant at a *p*-value < 0.05. GraphPad Prism 8.0 (GraphPad Software, Inc., California, USA) was used for statistical analysis.

## Results

### Expression of CD314 on Peripheral Circulating Cytotoxic CD8^+^ T-Cells

The activation marker CD314 was analyzed on cytotoxic CD8^+^ T-cells. There was no significant difference between the percentages of CD8^+^CD314^+^ T-cells in healthy controls and SLE-patients (98.7 ± 0.6% vs. 98.7 ± 1.3%, n.s.). There was also no significant difference between the percentages of CD8^+^CD314^+^ T-cells in SLE-patients with and without lupus nephritis and healthy controls, respectively (98.8 ± 1.1% vs. 98.4 ± 1.5% vs. 98.7 ± 1.3%, n.s.). Moreover, there was no significant difference between the percentages of CD8^+^CD314^+^ T-cells comparing active vs. inactive patients vs. healthy controls, respectively (98.3 ± 1.7% vs. 98.9 ± 0.9% vs. 98.7 ± 1.3%, n.s.).

### Decreased Percentages of CD8^+^CD107a^+^ T-Cells in SLE-Patients

**Figure 1:**
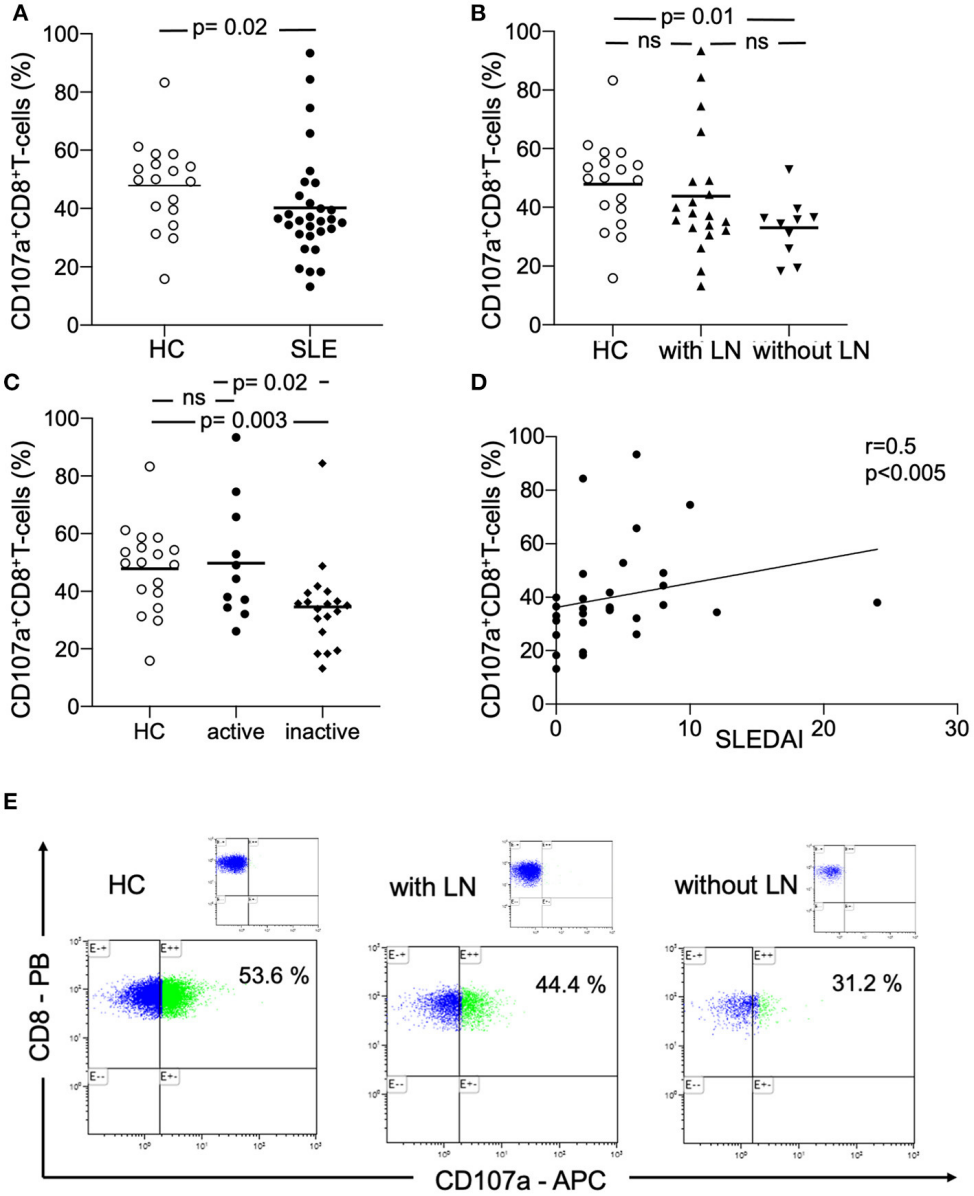
Peripheral circulating CD107a^+^CD8^+^ T-cells. **(A)** The percentages of CD107a^+^CD8^+^ T-cells in healthy controls (HC), SLE-patients (SLE), **(B)** patients with lupus nephritis (with LN) and without lupus nephritis (without LN) are shown. **(C)** The percentages of CD107a^+^CD8^+^ T-cells in healthy controls (HC), active SLE-patients (SLE) and inactive patients are shown. Frequencies of these T-cells are shown. Horizontal lines represent the mean. *P*-values were calculated using the non-parametric Mann-Whitney U-test. **(D)** Correlation between percentages of CD107a^+^CD8^+^ T-cells and disease activity (*n* = 30) as assessed by the systemic lupus erythematosus disease activity index (SLEDAI). Spearman analysis was performed to calculate the correlation. A *p*-value <0.05 was considered significant. **(E)** A representative dot plot of the flow-cytometry staining is shown for a healthy control (HC), a patient with lupus nephritis (LN) and without LN. The corresponding isotype control is illustrated.

The analysis of the cytotoxicity marker CD107a on CD8^+^ T-cells revealed a significant different expression (Figures 1A,E). The percentages were significantly decreased in SLE-patients (*n* = 30) as compared to healthy controls (*n* = 18) (40.2 ±18.5% vs. 47.9 ± 14.9%, *p* = 0.02). Next, the percentages were analyzed in SLE-patients according to renal involvement (Figure 1B). The percentages of CD8^+^CD107a^+^ T-cells were not different in SLE-patients without lupus nephritis as compared to lupus nephritis patients (33.0 ±10.1% vs. 43.8 ± 20.8%, n.s.). Interestingly, the percentages of CD8^+^CD107a^+^ T-cells were significantly decreased in SLE-patients without lupus nephritis as compared to healthy controls (33.0 ± 10.1% vs. 47.9 ±14.9%, *p* = 0.01).

### Amount of CD8^+^CD107a^+^ T-Cells Are Associated With Disease Activity in SLE

Percentages of peripheral circulating CD8^+^CD107a^+^ T-cells were analyzed in active and inactive SLE-patients (Figure 1C). Active SLE-patients had significantly increased percentages of CD107a^+^ cytotoxic T-cells as compared to inactive SLE-patients (49.8 ± 20.5% vs. 34.6 ± 15.1%, *p* = 0.02). There was also a significant difference between healthy controls and inactive SLE-patients (47.9 ± 14.9% vs. 34.6 ± 15.1%, *p* = 0.003).

There was a significant correlation between the percentages of peripheral circulating CD8^+^CD107a^+^ T-cells and disease activity assessed by SLEDAI (*r* = 0.5, *p* < 0.005, Figure 1D).

### Decreased Expression of CD8^+^CD107a^+^ T-Cells Is Associated With Immunosuppressive Treatment

**Figure 2:**
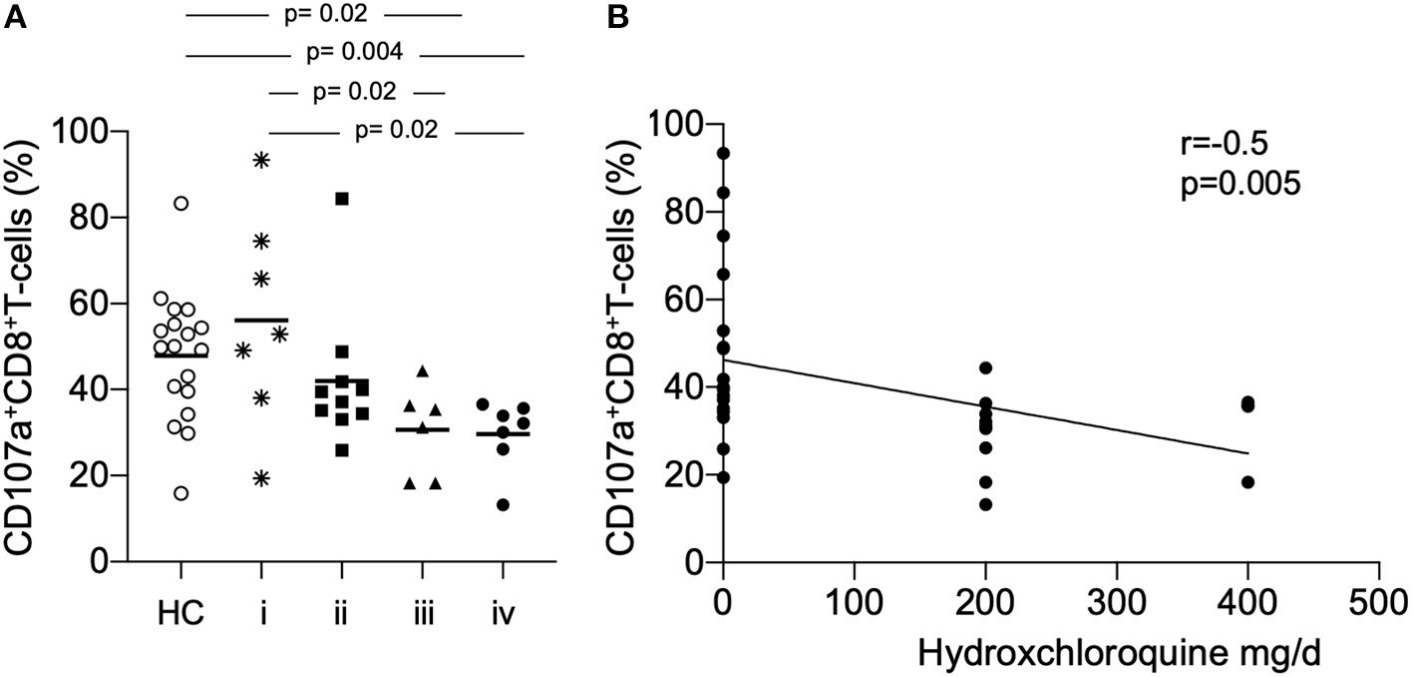
**(A)** The proportion of CD107a^+^ CD8^+^ T-cells we were analyzed according to the immunosuppressive treatment. Patients were subgrouped in (i) no treatment or prednisone alone (ii) prednisone and mycophenolate mofetil, azathioprine or cyclosporine (iii) prednisone and hydroxychloroquine (iv) prednisone and hydroxychloroquine combined with mycophenolate mofetil, azathioprine or cyclosporine. Patients were compared to healthy controls (HC). *P*-values were calculated using the non-parametric Mann-Whitney U-test. **(B)** Correlation between percentages of CD107a^+^CD8^+^ T-cells for all samples taken (*n* = 30) daily dose of hydroxychloroquine is shown. Spearman analysis was performed to calculate the correlation. A *p*-value < 0.05 was considered significant.

To assess the influence of immunosuppressive medication on the expression of CD107a^+^ we subgrouped patients in (i) no treatment or prednisone alone (ii) prednisone and mycophenolate mofetil, azathioprine or cyclosporine (iii) prednisone and hydroxychloroquine, respectively (iv) prednisone and hydroxychloroquine combined with mycophenolate mofetil, azathioprine or cyclosporine (Figure 2A). The analysis of CD107a on CD8^+^ T-cells showed a significant different expression in patients who received a combined treatment of prednisone and hydroxychloroquine as compared to prednisone alone or no treatment, respectively (30.6 ± 10.5% vs. 56.2 ± 24.4%, *p* = 0.02). The expression was almost similar in patients in group iv who received additionally mycophenolate mofetil, azathioprine or cyclosporine as immunosuppressive treatment (29.7 ± 8.0% vs. 56.2 ± 24.4%, *p* = 0.02).

There was no significant correlation between the daily dose of prednisone (mg/d) and the expression of CD107a on CD8^+^ T-cells. Remarkably, there was a significant negative correlation between the daily dose of hydroxychloroquine (mg/d) and the expression of CD107a on CD8^+^ T-cells (*r* = −0.5, *p* = 0.005, Figure 2B). Eleven patients were active on immunosuppressive treatment, six patients in group (i) were assessed with active disease.

### Renal Expression of CD107a Is Associated With Proteinuria

**Figure 3:**
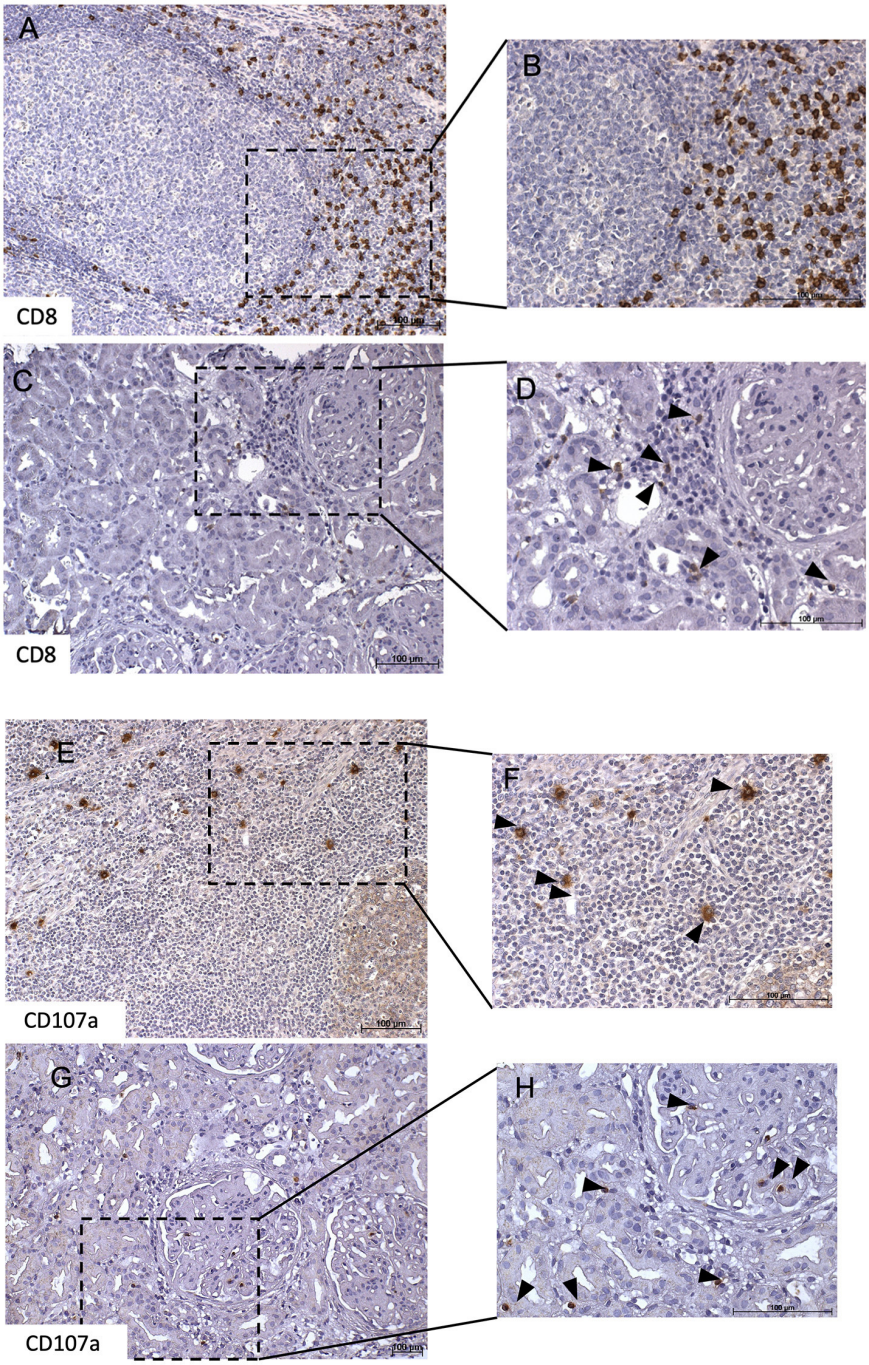
CD8^+^ and CD107a^+^ T-cell infiltrates in lupus nephritis. This figure shows representative immunohistochemical staining with anti-CD8 of a tonsil which served as positive control **(A,B)**. Immunhistochemical staining of a lupus nephritis renal biopsy shows an overview **(C)** with one glomerulum and interstitial lymphocytes. Several of these lymphocytes express CD8 as demonstrated in **(D)**. Next, representative immunohistochemical staining with anti-CD107a of a tonsil which served as positive control **(E,F)**. Immunohistochemical staining lupus nephritis renal biopsy shows an overview **(G)** with one glomerulum and interstitial lymphocytes. Several of these lymphocytes express CD107a as demonstrated in **(H)**.

**Figure 4:**
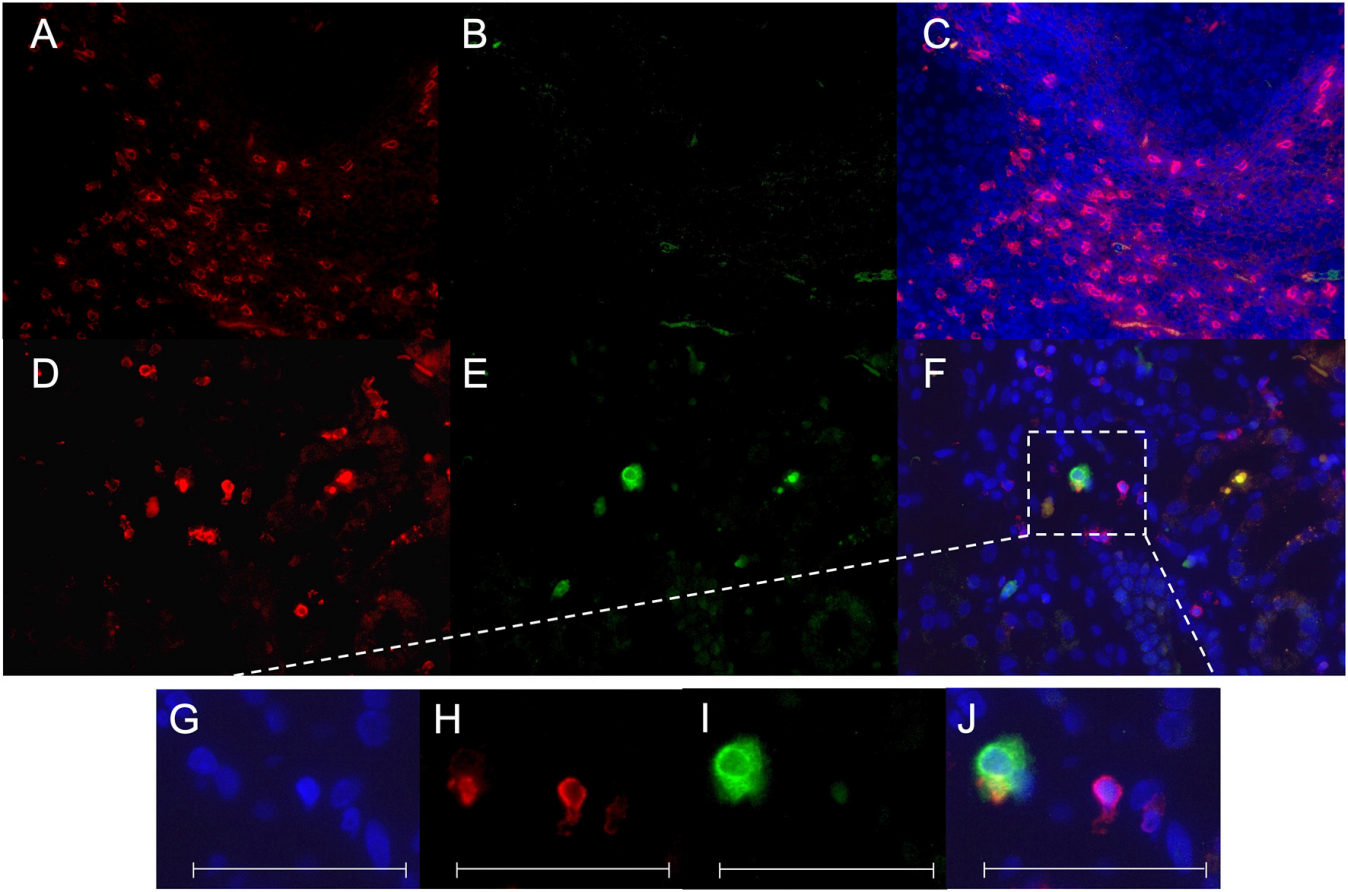
CD8^+^ CD107a^+^ immunofluorescence staining. A staining for CD8 Cy3 (red), CD107a FITC (green) and colocalization of CD8/CD107a/DAPI was performed in a tonsil as positive control **(A–C)** and a representative renal biopsy of an SLE patient with lupus nephritis (WHO class IV) **(D–F)**. A magnification of a double-positive CD8^+^CD107a^+^ kidney infiltrating cell is shown in **(G–J)**. All scales represent 100 μm.

**Figure 5:**
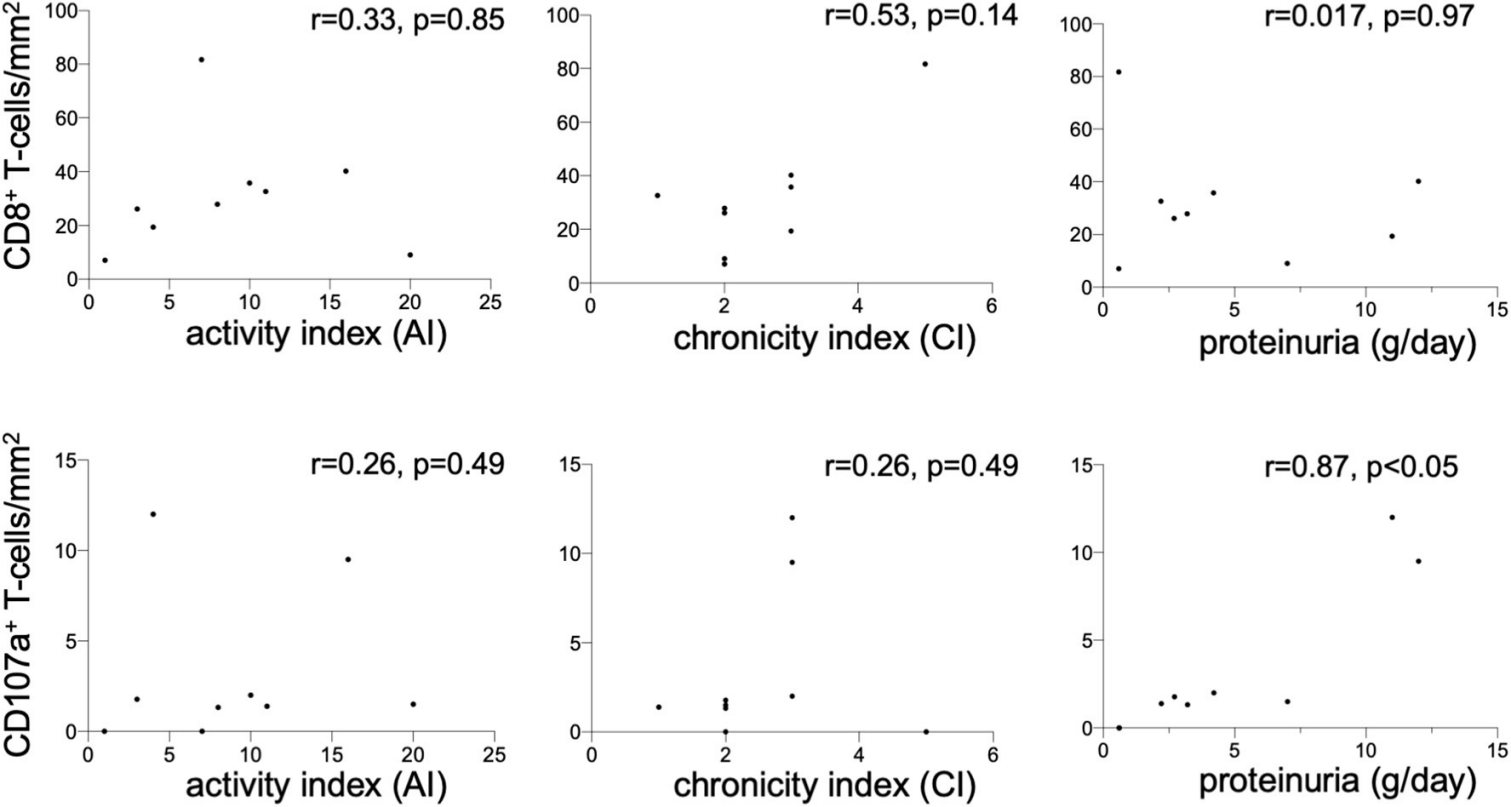
Renal CD8^+^ and CD107a^+^ T-cells. Correlation between the CD8^+^ T-cell count (cells/mm^2^) in renal biopsies of nine lupus nephritis patients and renal histopathology parameters activity index (AI), chronicity index (CI) and proteinuria (g/d). The same correlation was performed for CD107a^+^ T-cell count (cells/mm^2^) in renal biopsies. Spearman analysis was performed to calculate the correlation. A *p*-value < 0.05 was considered significant.

Immunohistochemical CD107a staining of renal biopsies of SLE-patients showed a mean count of 59 ± 22.6 CD8^+^-cells/mm^2^ (Figure 3). The highest amount of 30.9 ± 20.9 CD8^+^-cells/mm^2^ where present in the extraglomerular compartment and only very few cells 0.1 ± 0.2 CD8^+^-cells/mm^2^ could be detected intraglomerular. CD107a expression could be detected on 3.7 ± 2.7 CD8^+^-cells/mm^2^. A cell count of 2.0 ± 2.4 CD107a^+^-cell/mm^2^ was extraglomerular and 0.3 ± 0.4 CD107a^+^-cells/mm^2^ intraglomerular. Double-positive cell were found in extraglomerular infiltrates (Figure 4). The intrarenal cell count of CD8^+^-cells and CD107a^+^-cells correlated with the activity and chronicity index (Figure 5). There was no significant correlation between cell counts and the activity or chronicity indices. The degree of the proteinuria was significantly correlated with intrarenal cell count of CD107a^+^-cells (*r* = 0.87, *p* < 0.05).

## Discussion

Activated cytotoxic T-cells exert their effector function mainly by release of granzyme B and perforin. This release is dependent on cell-cell interaction. Ligation of CD107a (LAMP-1) has been described as a pivotal axis which leads to CD8^+^ T-cell activation. CD107a is a marker of degranulation of cytotoxic NK and CD8^+^ T-cells (17).

The present study demonstrates a decreased expression of CD107a on CD8^+^ T-cells SLE-patients as compared to HC. The decreased proportion of CD107a^+^CD8^+^ T-cells was especially found in SLE-patients without lupus nephritis. This might be explained by a lower disease activity in this group because correlation with SLEDAI showed a significant correlation. This significant finding was confirmed in a previous observation by Holcombe et al. The authors reported a significant correlation between LAMP-1 expression on peripheral mononuclear blood cells of SLE-patients with disease activity assessed by Systemic Lupus Activity Measure (SLAM) but not with SLEDAI (10). The different results regarding the activity scores are most likely due to the different patients cohorts included. In our study predominantly patients with lupus nephritis were included. These patients have frequently higher activity scores in comparison to patients without lupus nephritis. Moreover, renal disease activity is considered with more items in the SLEDAI than in the SLAM resulting in different correlations (18). Besides, the study by Holcombe et al. recruited 10 of 46 patients without immunosuppressive medication and even more important for the data interpretation LAMP-1 expression was determined on PBMCs in contrast to specific subsets such as CD8^+^ T-cells. This resulted in very low expression levels of 1.33 ± 0.25%).

*Ex vivo* experiments have shown that isolated PBMCs had an increase of CD107a expression in the presence of phytohemagglutinin (PHA) in a dose dependent manner. The induction peaked 30 min. after stimulation suggesting that rapid cell surface expression is due to translocation from intracellular vesiculars which are a major reservoir of LAMP proteins (9). Stimulation with IL-2 has been reported to be also a potent stimulus to trigger CD107a expression on NK and CD8^+^ T-cells which was associated with increased cytotoxicity (17). Another study demonstrated that the surface molecule signaling lymphocytic activation molecule family member 4 (SLAMF4; CD244) is pivotal for the cytotoxic activity of CD8^+^ T-cells assessed by CD107a in SLE-patients (19). CD8^+^ T-cells of SLE-patients with selective loss of SLAMF4 showed a decreased CD107a expression upon stimulation with an anti-CD3 antibody for 2 h.

In patients with Churg-Strauss-syndrome the expression of CD107a was significantly increased after polyclonal stimulation with anti-CD3 (20).

Remarkably, the expression CD107a on unstimulated CD8^+^ T-cells was relatively low in the study by Aktas et al. In contrast to our study isolated peripheral mononuclear blood cells were used which could influence the expression. In the presence of IL-10 there was no significant increase in CD107a expression on CD8^+^ T-cells (17). Thus, CD107a expression seems to be dependent on the surrounding cytokine milieu which might be *in vivo* variable during the course of disease in SLE-patients.

These studies support the idea that CD107a indicates T-cell activation. Besides, the lysosomal-associated membrane proteins (LAMPs) appear on the cell surface after exocytosis of cytotoxic granules. Thus Cohen et al. hypothesized that CD107a is transiently protecting cytotoxic lymphocytes from self-destruction (21).

The migration of cytotoxic T-cells to the kidneys and target organs during inflammation is a frequently reported observation. In this light the present finding of CD8^+^ cells in renal biopsies of SLE-patients are confirmative. However, data on cytotoxic activity of CD8^+^ T-cells in these biopsies are scarce. Thus, we stained CD107a cells in kidney biopsies. The presence of intrarenal CD107a cells was significantly correlated with proteinuria. This might indicate that these cells were activated and recently degranulated. A Denys-Drash murine model of nephrotic syndrome established to determine if lysosome activity in proteinuric mice demonstrated increased glomerular staining of LAMP-1 as compared to wild type mice (22). In accordance with our observation Carson and coauthors reported an association between LAMP-1 expression and degree of proteinuria. The present study provides evidence that CD107a is lower expressed on T-cells in SLE or downregulated by immunomodulating therapy, respectively. Interestingly, the effect diminishes during active disease. This might be explained by general disease activity or more likely by renal activity as well. The observation of relatively high CD107a expression in healthy controls in our cohort is not fully elucidated. Interestingly, the expression in our healthy control group was comparable with healthy non-pregnant women in a recent report (23). Exhaustion of cytotoxic T-cells in SLE-patients could be a possible explanation.

Moreover, CD107a has been described to mediate cell adhesion to vascular endothelium which potentially enables T-cell migration into kidney during active lupus nephritis (9). In *in vitro* experiments with stimulated NK cells from patients with granulomatosis with polyangiitis (GPA) demonstrated directly killing capacities of CD107a^+^ NK cells of renal microvascular endothelial cells (24).

Immunosuppressive treatment might have an additional influence on cytotoxic activity and degranulation. Our data suggest that common immunosuppressive treatment could inhibit cytotoxic activity of CD8^+^ T-cells by decreasing CD107a^+^ expression. Interestingly, hydroxychloroquine is the agent which is most likely responsible since a strong negative correlation with the daily dose was demonstrated. A possible explanation could be the disruption of lysosomes by hydroxychloroquine which has been indicated by *in vitro* exposure of rat hepatocytes in co-culture experiments (25).

In conclusion, the present data suggest a critical role of CD107a for CD8^+^ T-cell activation in particular in active disease. The detection of CD107a^+^CD8^+^ T-cells in lupus nephritis biopsies highlights the most likely effector cell function in renal involvement. The lack of functional experiments with renal infiltrating T-cells which remains technically very difficult is a limitation of this study. Nevertheless, there is a growing body of evidence that CD107a^+^ might be a future therapeutic target to address cytotoxic T-cells.

## Data Availability Statement

The raw data supporting the conclusions of this article will be made available on request to the corresponding author.

## Ethics Statement

The studies involving human participants were reviewed and approved by Ethical Board University Hospital Essen. The patients/participants provided their written informed consent to participate in this study.

## Author Contributions

AW collected the samples, performed the experiments and the statistical analysis. BW participated in research design, participated in the acquisition and analysis of the data and in the writing of the manuscript. BT and KL participated in the acquisition and analysis of the data. KA participated in the acquisition and analysis of the data and in the writing of the manuscript. AK drafted the manuscript. WA participated in the analysis of the data and drafted the manuscript. OW participated in the performance of the research, in research design and in the writing of the paper. SD designed the study, collected clinical data, analyzed the data and drafted the manuscript. All authors contributed to the article and approved the submitted version.

## Conflict of Interest

The authors declare that the research was conducted in the absence of any commercial or financial relationships that could be construed as a potential conflict of interest.
